# Assessing ad-hoc adaptations’ alignment with therapeutic goals: a qualitative study of lay counselor-delivered family therapy in Eldoret, Kenya

**DOI:** 10.1186/s43058-023-00477-5

**Published:** 2023-08-29

**Authors:** Bonnie N. Kaiser, Julia Kaufman, Jonathan Taylor Wall, Elsa A. Friis Healy, David Ayuku, Gregory A. Aarons, Eve S. Puffer

**Affiliations:** 1 Department of Anthropology; Global Health Program, University of California San Diego, La Jolla, CA USA; 2 Duke Global Health Institute, Durham, NC USA; 3 Department of Psychology & Neuroscience, Duke University, Durham, NC USA; 4 Department of Behavioral Sciences, School of Medicine, College of Health Sciences, Moi University, Eldoret, Kenya; 5 Department of Psychiatry, University of California San Diego, La Jolla, CA USA; 6 ACTRI Dissemination and Implementation Science Center, University of Cailfornia San Diego, La Jolla, CA USA

**Keywords:** Ad hoc adaptations, Task-shifting, Family functioning, Kenya

## Abstract

**Background:**

A key question in implementation science is how to balance adaptation and fidelity in translating interventions to new settings. There is growing consensus regarding the importance of *planned adaptations* to deliver interventions in contextually sensitive ways. However, less research has examined *ad-hoc adaptations*, or those that occur spontaneously in the course of intervention delivery. A key question is whether ad-hoc adaptations ultimately contribute to or detract from intervention goals. This study aimed to (a) identify ad-hoc adaptations made during delivery of a family therapy intervention and (b) assess whether they promoted or interrupted intervention goals.

**Methods:**

*Tuko Pamoja* (Swahili: “We are Together”) is an evidence-informed family therapy intervention aiming to improve family dynamics and mental health in Kenya. Tuko Pamoja employs a task-shifting model, delivered by lay counselors who are afforded a degree of flexibility in presenting content and in practices they use in sessions. We used transcripts of therapy sessions with 14 families to examine ad-hoc adaptations used by counselors. We first identified and characterized ad-hoc adaptations through a team-based code development, coding, and code description process. Then, we evaluated to what extent ad-hoc adaptations promoted the principles and strategies of the intervention (“TP-promoting”), disrupted them (“TP-interrupting”), or neither (“TP-neutral”). To do this, we first established inter-coder agreement on application of these categories with verification by the intervention developer. Then, coders categorized ad-hoc adaptation text segments as TP-promoting, TP-interrupting, or TP-neutral.

**Results:**

Ad-hoc adaptations were frequent and included (in decreasing order): incorporation of religious content, exemplars/role models, community dynamics and resources, self-disclosure, and metaphors/proverbs. Ad-hoc adaptations were largely TP-promoting (49%) or neutral (39%), but practices were TP-interrupting 12% of the time. TP-interrupting practices most often occurred within religious content and exemplars/role models, which were also the most common practices overall.

**Conclusion:**

Extra attention is needed during planned adaptation, training, and supervision to promote intervention-aligned use of common ad-hoc adaptation practices. Discussing them in trainings can provide guidance for lay providers on how best to incorporate ad-hoc adaptations during delivery. Future research should evaluate whether well-aligned ad-hoc adaptations improve therapeutic outcomes.

**Trial registration:**

Pilot trial registered at clinicaltrials.gov (C0058)

Contributions to the literature

- There is evidence that ad-hoc adaptations, or those that occur spontaneously in the course of intervention delivery, occur more often than planned adaptations. Yet, there is significantly more literature examining planned adaptations and their effects on intervention fidelity.
- Evidence is mixed on whether ad-hoc adaptations tend to contribute to intervention effectiveness or undermine it.
- We systematically studied alignment of ad-hoc adaptations with intervention goals and found they tended to align with intervention goals far more often than interrupting goals.
- We describe how to incorporate discussion of ad-hoc adaptations into training and supervision to promote their use in intervention-aligned ways.

## Introduction

A key question in implementation science has been how to balance the seemingly competing needs of maintaining fidelity to an evidence-based practice (EBP) and achieving fit to local contexts [13, 14, 30, 39, 50]. Although there are examples of well-documented adaptations (e.g., [3, 16]), such adaptation processes tend not to be recorded sufficiently or systematically [7]. Increasingly, theories, frameworks, and processes are being articulated to guide processes of cultural and contextual adaptation to help fill this gap [2, 21, 22, 26, 32, 38, 51, 53]. There is also growing consensus that “adaptation happens” and implementers should focus on ensuring that core intervention functions are preserved, while using adaptations to improve contextual fit [15, 17, 27, 30].

Global mental health is a particularly relevant arena for addressing these questions, as cultural adaptation has been a concern for global mental health for decades [8, 9, 11, 24, 45]. Earlier concerns have been whether the understanding and etiology of mental health disorders are similar enough across contexts to allow for transfer of mental health treatments developed almost exclusively in high-income countries [20]. Results are mixed, but most literature suggests that transfer is possible, with caveats about the need for adaptation to content, language, and presentation methods [25]. These considerations are important for individual psychotherapies and perhaps even more so for family-level interventions given strong cultural and contextual influences on family structure and roles and variability in which family interaction styles are most important and effective in a given context [32, 36]. Despite this, examples of cultural adaptation for parenting or family interventions are relatively sparse compared to individual psychotherapies [5–7, 31, 32, 40, 42].

Also influencing adaptation processes is the use of task-shifting models in global mental health—training non-specialist workers to deliver mental health treatments. Task-shifting has become a widely used strategy for disseminating mental health treatments in low- and middle-income countries given the scarcity of human resources and large treatment gap [4, 28, 48]. Evidence is building that mental health treatments can be effectively implemented by non-specialist workers [11, 18, 20, 41, 46]. Training non-professionals also brings a more diverse group of community members and community-based perspectives into mental healthcare, leading to both opportunities and challenges related to adaptations. As task-shifting interventions expand, there are increasing efforts to improve contextual sensitivity and cultural fit of interventions while balancing fidelity to original models [5, 10, 35].

Despite growing consensus regarding the importance of adaptation to ensure that interventions are delivered in contextually sensitive ways, the vast majority of literature on adaptation—in both global mental health and implementation science—has focused on *planned adaptations* [33, 38]. For example, in a systematic review of adaptations of evidence-based psychotherapies, Wiltsey Stirman and colleagues [52] found over 100 eligible articles, all of which addressed planned adaptations only. The authors concluded that overall, these adaptations did not seem detrimental to the EBPs. Less research has examined *ad-hoc adaptations*, or those that occur spontaneously in the course of intervention delivery, despite some evidence that they occur more often than planned adaptations [38]. Studies have begun to document and evaluate ad-hoc adaptations and have concluded that they can ultimately contribute to intervention effectiveness but could also undermine it [5, 23, 38].

The intervention described herein—a family therapy intervention in peri-urban Kenya—is particularly appropriate for studying ad-hoc adaptations. Many task-shifting interventions train primary care providers or community health workers, who are overburdened with work. In contrast, our study countered this concern by identifying and training individuals who already spend time providing informal care in the community [44]. Although counselors are trained in effective and structured pedagogical and therapeutic strategies, they are afforded flexibility in how they deliver the intervention, leaving room to implement ad-hoc adaptations, such as pedagogical practices and ways of explaining content that are not part of the manualized intervention. This qualitative study also builds the somewhat small literature on adaptation of interventions involving and targeting the interaction patterns within the whole family system. We aimed to (a) identify ad-hoc adaptations made during delivery of a family therapy intervention in Kenya and (b) assess whether these adaptations promoted or hindered intervention goals. A broader goal is to assess our approach to identifying and evaluating ad-hoc adaptations and reflect on how it could be applied in other settings.

## Methods

### Setting

This study took place in two peri-urban communities near Eldoret, Kenya, located in Uasin Gishu county in the Western part of the country. Eldoret had a population of 475,659 in 2019 and is growing quickly [49]. Mental, neurological, and substance use disorders account for 10% of the disease burden in Uasin Gishu County, with depression accounting for the highest burden [27]. Kenya is recently addressing its shortage of mental health workers: for its population of almost 52 million, Kenya has approximately 8000 mental health professionals (15.32 per 100,000 population, compared to 0.18 per 100,000 in 2017 [37].

In the city of Eldoret, Moi Teaching and Referral Hospital provides some psychiatric care services, including inpatient care and limited outpatient care. Moi University is also located in this area and provides undergraduate training in medical psychology. The available psychiatric care services and psychology training focus mostly on treatment for adults with serious mental illness, and very little family-specific or child-focused training or treatment is available.

### Intervention

*Tuko Pamoja* (Swahili: “We are together”) is a family therapy intervention being delivered in Eldoret [44]. Tuko Pamoja is designed to decrease family conflict and improve family functioning in order to improve caregiver and child mental health and reduce children’s risk behaviors. It is modular and includes components of solution-focused, family systems, and cognitive-behavioral therapies [12, 19, 47]. These practices were selected based on qualitative data that suggested they best fit cultural norms and needs.

Planned integration of practices and adaptations to improve fit to family processes in the Kenyan context included providing context-specific examples depicting interaction patterns described in formative work. Core elements were also structured and simplified into specific steps for lay providers [44]. However, because Tuko Pamoja was designed with transferability in mind, its structure emphasizes and encourages flexibility, such as using appropriate clinical skills while allowing additions that aim to make the intervention more understandable, comfortable, and applicable to the family within this context. See Table 1 for a summary of core process components and Table 2 for more information on Tuko Pamoja design and counselor training and supervision.

**Table 1:** Core process components of Tuko Pamoja

Focus on the family as a system (connected, dynamic)
Involvement of all family members
In-session skills practice, emphasizing communication
Focus on goal setting and solutions identified by the family
Focus on behavior change
Assessment and tracking of distress, change, and progress

**Table 2:** Intervention design, training, and supervision

Tuko Pamoja (Kiswahili: “We are together”) is a family therapy intervention developed based on exploratory qualitative research with Kenyan families, mental health providers, and informal counselors identified by communities [43]. The exploratory research indicated primary areas of focus for an intervention, including conflict related to roles and responsibilities and avoidant or negative communication during problem-solving and decision-making. Based on these findings, elements of multiple evidence-based practices (EBPs) were incorporated based on their alignment with the identified needs and existing local counseling practices: (1) solution-focused family therapy, (2) systems-focused approaches, (3) parenting skills training, including behavior management and positive relationship-building, and (4) cognitive behavioral therapy. Tuko Pamoja is modular, with families receiving only those modules relevant to them (marital, parent-child, whole family). Each module is completed over the course of 2 to 5 counseling sessions, with treatment lasting approximately 12 to 15 sessions [44]. Strategies drawn from the above EBPs were combined and streamlined for delivery by lay counselors, allowing them to be distilled into concrete “steps” with succinct manualized instructions. Though the manual is structured, with specific steps and goals within a module, activities are not time-limited, and lay counselors are provided flexibility in delivery [34], with a key goal being active participation of family members, in-session communication, and generation of solutions by families. To best fit existing practices in communities, Tuko Pamoja draws on “natural counselors” – people who already provide informal counseling within their communities (e.g., pastors, community leaders; [43]). The goal was to provide new skills to people who are already sought out by families for advice and conflict mediation, without significantly expanding their workload. This study draws on data from a pilot of Tuko Pamoja that included 8 counselors, with 2 being a husband-wife team who counseled together (see Table 3). These natural counselors were nominated by community leaders then interviewed by the research and implementation team to assess interest, availability, and the extent to which delivering Tuko Pamoja would fit into their existing informal counseling routines. Each counselor typically only had one to two case families during this study, and counselors recruited families themselves, whom they knew and believed would benefit. Counselors received a 10-day training that included training in non-specific clinical skills (e.g., reflection, validation) and process skills central to family treatment and then training on manualized content. Training included didactic instruction and demonstrations of intervention steps, followed by extensive role play, coaching, and feedback. Counselors were also trained on how to identify safety concerns, including concerns related to suicide and violence, and the process for consulting with supervisors to address them with safety planning and referrals. A total of 14 families were included in the study, with 10 completing therapy. With the exception of one counselor who worked with 3 families and another counselor who worked with only one family, all other counselors worked with two families who completed part or all of the intervention. We used a tiered supervision process with local supervisors who were students in medical psychology at a local university and expert consultation by US and Kenyan psychologists. Local supervisors completed a 4-day training on basics of the intervention, as well as participating in the 10-day counselor training [43]. Supervision occurred after each therapy session, either by phone or in person. Supervisors listened to recordings of therapy sessions and elicited counselors’ impression of sessions, practiced collaborative decision-making to address challenges, and used role play to support development of clinical skills. The uniquely intensive practice of listening to each session allowed supervisors to flag any instance of a counselor using practices that were not aligned with TP content and principles. These were then addressed directly by (a) guiding the counselor to understand the disconnect and, when relevant, potential for harm and (b) developing and role playing a plan to course correct during the next session.	

### Data and analysis

This study draws on qualitative data collected as part of a pilot study of Tuko Pamoja in 2015–2016 (see Tables 2 and 3). All sessions were audio-recorded in Swahili and transcribed into English by the supervisor assigned to that counselor. We randomly selected six transcripts from each of the ten families who completed therapy. We coded all transcripts from the four families that had fewer than six therapy sessions (two families had 3 sessions; one each had 1 and 2 sessions), for a total of 69 transcripts. While formal coding and analysis were completed by a team of US-based researchers, the process was informed throughout by Kenyan team members. Clinical supervisors were all Kenyan, and they noted when there was cultural or context-specific material that counselors presented in the sessions. These materials were then discussed with the US-based supervisor, including discussion of how they fit into the therapy process. These discussions and insights informed how we conceptualized ad-hoc adaptations in this study, evaluated fit with the intervention, and interpreted the findings.

**Table 3:** Characteristics of counselors during pilot study of Tuko Pamoja in 2015–2016

Characteristic	Counselors (*n* = 8)^*^
Gender (male)	50%
Age range (mean)	36–53 (45)
Religion	
Christian	7
Muslim	1
Education	
Some/completed primary	3
Some secondary	3
Diploma	1
Vocational	1
Occupation	
Church/mosque leader	6
Other community leader	1
Counselor	1

^*^There was one husband–wife couple who counseled families together

We used thematic analysis to identify and describe ad-hoc adaptations by counselors, or ways that counselors drew upon their own local understandings and norms and introduced non-manualized material into the therapeutic process. We used an inductive approach to theme identification, reviewing transcripts from different families and counselors in order to identify patterns and noting potential themes. We developed these into codes, then iteratively edited and added to the initial list of codes and definitions by reviewing additional transcripts until saturation was reached (i.e., no new themes emerged from additional data). We independently coded three transcripts, compared our coding, edited code definitions, and repeated until we reached 80% inter-coder agreement. Two researchers each coded half the transcripts using NVivo, with all transcripts for a given family assigned to the same coder. Of the *n* = 69 transcripts, 4 had no ad-hoc adaptations.

We then reviewed all coded segments and categorized them based on how their use related to intervention goals. “TP-promoting” reflected that a practice was used in line with and supported intervention goals; “TP-interrupting” referred to practices that hindered or interrupted intervention goals (e.g., providing concrete solutions rather than allowing the family to do so); “TP neutral” were practices that were neither promoting nor hindering intervention goals (e.g., “lectures” that were not inconsistent with the intervention but were long-winded, limiting the clients’ opportunities for engaging). This is consistent with Moore and colleagues’ [38] categories of positive, negative, or neutral valence. Supervised by the Tuko Pamoja developer, three researchers completed an inter-coder agreement exercise using several transcripts before independently coding remaining transcripts. The developer then reviewed all second-round coding to ensure accuracy.

We developed summaries of each code, or category of ad-hoc adaptation, including descriptions of their use in TP-promoting, TP-interrupting, or neutral ways. We compared our findings to existing taxonomies [1, 38] to help categorize and describe the types of ad-hoc adaptations found. We then quantified how often each practice was applied in TP-promoting, TP-interrupting, or neutral ways. Using coded segments (*n* = 209) as the unit of analysis, we calculated an “alignment proportion” for each practice, representing the ratio of TP-promoting to TP-interrupting applications of the practice.

## Results

**Fig. 1:**
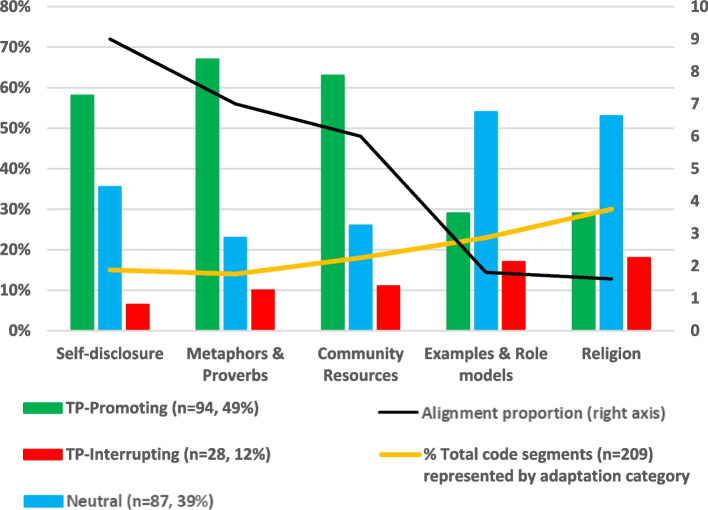
Ad-hoc adaptations’ alignment with intervention goals

Lay counselors integrated a diverse array of ad-hoc adaptations. From most to least frequent, these included (1) religion, (2) examples and role models, (3) incorporating community resources and dynamics, (4) self-disclosure, and (5) metaphors and proverbs. These practices have been described in greater detail elsewhere [29]. Here, we briefly summarize each practice and present findings regarding fit with intervention goals. Overall, these practices tended to be used in ways that aligned with intervention goals far more often than they interrupted intervention goals (Fig. 1). However, the most frequently used practices (Religion and Examples/Role models) were applied in ways contradictory to intervention goals about one-fifth of the time.

### Religion

Almost all counselors suggested faith and prayer as important strategies for coping with challenges that families faced. One counselor (a local community leader), plus the husband–wife counseling pair, who were church leaders, accounted for most instances of religious ad-hoc adaptations. These counselors frequently emphasized the importance of the family attending church together to be united and successful as a family (see “Community Resources”). Beyond religiosity as a solution, references to religion, praying, going to church or mosque, God, the Bible, or Jesus were ubiquitous in most counselors’ therapy. Counselors often referenced putting hope or trust in God, frequently using phrases like “God willing” and “pray to God.” Counselors and families usually prayed together before and after therapy. These practices seemed to align with families’ perspectives and preferences, as family members often brought up religion during sessions and invoked faith in God as an important part of their lives.

Where counselors’ ad-hoc incorporation of religion best aligned with intervention goals, it was through skillful use of references to God or prayer to affirm and recognize families’ progress and encourage them to continue working hard. Such examples were often framed by the counselor as “I thank God because,” followed by mentioning a concrete form of progress they had noted. Additionally, these adaptations often encouraged the family to be active in creating change, rather than passively relying on God. For example, one counselor incorporated gratitude to God with reminders that only the couple can improve their marriage (see Table 4). Additionally, counselors validated families’ goals by praying for families during sessions. Finally, counselors effectively incorporated Biblical or Koranic passages to promote intervention concepts, for example:

*Jesus asked his disciples, “Between these two, which one can withstand the storm? The one on the sandy ground or the one on the rocky ground?” So, families are required to be built on the rock, on firm ground.”* (Family 11)

**Table 4:** Summary of ad-hoc adaptation types and their alignment with intervention goals

Ad-hoc adaptation type	Description	Alignment with intervention goals	Example
Religion	• Faith, prayer suggested as strategies for coping with challenges • Religious references (church/mosque, Bible) common for most counselors • Encouraged to put trust in God • Counselors, families prayed together • Used religious texts to promote intervention content	• Mostly neutral but TP-interrupting 1/5 time • TP-promoting: References to God, prayer to affirm and encourage families’ progress; encourage families to actively create change, rather than passively rely on God • TP-interrupting: Tell families what to do (e.g., prayer as only solution), sometimes with religious justification	“I thank God because you have reached your goal. […] My prayer for you is that you will be able to build that goal to be the good foundation for you because you have decided on a good goal and will improve you in your marriage.” (Family 10)
Metaphors and proverbs	• Metaphors used to explain intervention content	• Mostly TP-promoting; only TP-interrupting in 10% instances • TP-promoting: Metaphors effectively interwoven with eliciting problems, solutions or affirming family • TP-interrupting: Sometimes become long lectures that prevent family from contributing, developing solutions	“When you want to plant vegetables, you can plant just a little bit of the vegetables, but you will harvest a lot from that. And so, what will you do in order to start this love, so that it can continue to flourish and grow?” (Family 12)
Incorporating community resources and dynamics	• Institutions (e.g., schools, businesses) and individuals (e.g., elders) discussed in relation to both possible help and harm (e.g., neighbors causing issues) • Counselors encouraged families to seek out resources as potential emotional, problem-solving supports or material supports (e.g., for loans) • Cultural dynamics rarely referenced; emphasized alignment of intervention, cultural values	• Mostly TP-promoting; only TP-interrupting in 10% instances • TP-promoting: Encourage families’ active identification of solutions • TP-interrupting: Tell families what to do; over-promise; become judgmental	“We say respect is powerful in Nandi; our people were not fools because you can see all these things that we use [in this program] are the things that our people used to do. [...] We need to consider it and put together our culture and what we have now.” (Family 1)
Examples and role models	• Examples of people in similar situations as families. Usually real people family or counselor knows. Sometimes famous people or hypothetical examples • Mostly positive stories used to motivate; some negative examples used to warn • Examples from previous families counseled to establish expertise, instill hope	• Mostly neutral but TP-interrupting 1/5 time • TP-promoting: Encourage families through success stories or gentle warnings, while emphasizing necessity of effort; validate shared experiences • TP-interrupting: Overstate possible improvements; examples sometimes long, confusing; offer solutions instead of family developing them; negative examples sometimes foreboding, heavy handed	“I had a [neighbor] and had the same problem as yours. They were quarreling and fighting all the time, and I had to sit down with them, and we talked, and they both listened to me, and we managed to solve their problem. […] Misunderstanding in a family is normal, but you have to look beyond that and see the future of your children.” (Family 15)
Self-disclosure	• Counselors shared own experiences (e.g., problem drinking, relationships, emotions) • Sometimes hypothetical examples of how they would handle similar situation • One of least frequent but used by widest range of counselors	• Most intervention-aligned type; large minority of instances neutral • TP-promoting: Validate experiences; model active solutions; promote communication • TP-interrupting: Become lecturing or judgmental; promote harmful behaviors	“Do not think we [counselors] do not have problems; we also have problems. I also love this program because it has helped me; when I pass through such challenges, I know that I need to do this and this.” (Family 1)

In contrast, there were several uses of religion in ways that disrupted core intervention strategies and, in some cases, were likely harmful, most coming from one counselor. The primary way was telling families what to do by being specific and forceful, rather than encouraging their own efforts or solutions—such as suggesting prayer as the *only* solution. In another extreme instance, a counselor used references to God and the Bible to admonish a wife to sleep with her husband despite his heavy drinking: “*[Wife], are you fulfilling what God wanted you to do?”* (Family 4). In response to such admonitions, the wife protested that her husband should first quit drinking and then she will sleep with him, but the counselor continued pushing. A related problem was over-promising or guaranteeing, such as giving examples of children from the community who received funding for school fees or succeeded in school because they loved the church. All of these adaptations tended to block discussion of how the family could take their own steps or explore their own solutions, which are central aspects of therapy. Notably, counselors who often used religious content did so in a combination of TP-promoting, neutral, and interrupting ways; we did not see strong evidence of certain counselors using the strategies uniformly positively or negatively.

Most of the time, religious ad-hoc adaptations neither promoted nor interrupted intervention goals. Several counselors encouraged family members to pray or turn to God as a solution: “*When you put all your thoughts to God praying as you sleep, pray all the time, night, you will see*” (Family 1). Sometimes counselors used personal testimony (see “Self-disclosure”), describing how God helped them overcome challenges such as alcoholism. Counselors sometimes framed families’ problems in religious terms, for example “*You as parents have a big role of defeating Satan*.” (Family 4). Finally, counselors used passing references to God, such as saying “I thank God” when they reported doing well. Overall, these uses of religion reflect somewhat less skillful attempts to convey intervention goals, oftentimes suggesting solutions rather than encouraging families to do so, or emphasizing the role of prayer or God without actively encouraging families to work to improve their relationships. At the same time, some of these adaptations could coexist alongside TP strategies without interrupting or contradicting them.

### Metaphors and proverbs

Counselors used metaphors^1^ and proverbs to explain the program’s concepts to families and to support counseling strategies. This was the second most aligned practice in proportion of intervention-promoting to intervention-interrupting content. Common metaphors included building a house to represent solving family problems and relating the family to a body or machine, with family members being parts of a whole. For example, one counselor related therapy to “filling in holes” of a house: “*As the Swahili saying goes; ‘If you don’t repair a crack on the wall, you will build a new wall.’*” (Family 4). Additionally, several metaphors related to farming (see Table 4).

For the most part, metaphors were effectively interwoven with eliciting problems and solutions or affirming the family, emphasizing the need for them to do the work of repair, for example, “*The most important thing is that you are the ones to build this house*” (Family 4). At other times, metaphors themselves were appropriate but were undermined when presented as part of long lectures that did not allow the family to interact or contribute. A few metaphors had unclear messages. It is possible that this was due to issues with translation and that the families could understand and interpret them, but several times, counselors didactically told stories that were long-winded and interrupted the therapeutic interaction.

Some metaphors were delivered slightly judgmentally, for example, in this metaphor addressing a father’s substance use:

*In our culture, they say that you blame the eagle together with the hen. […Alcoholism] has destroyed the family; first it has taken away family life; secondly it has destroyed the economy of the family*” (Family 4)

While most counselors incorporated metaphors and proverbs, two-thirds of coded segments were from the husband–wife counseling pair, including the only TP-interrupting instances.

### Community resources and dynamics

Counselors sometimes mentioned community resources and cultural dynamics as they related to families’ problems and potential solutions. Community resources include institutions such as hospitals, churches, police, schools, and businesses, and individuals such as neighbors, chiefs, elders, and the counselors themselves. Resources were discussed in terms of both ways they might help and harm people. Counselors named these community resources to encourage families to seek them out, praise their current engagement, or elicit resources they could draw upon. Resources were referenced as potential emotional supports, to help with problem-solving—particularly elders or extended family members—or material supports such as providing loans, collective fundraising, or bursaries. Almost all counselors referenced such resources, though one counselor, who worked with a family with both financial and medical needs, accounted for half of the instances.

Church and medical care were the most frequently named institutions, though these were mostly by two counselors. For example, discussions about HIV testing arose often after one adolescent disclosed a rape. On another occasion, a counselor offered to take a mother to a nearby hospital for counseling services because she had expressed suicidal thoughts. In contrast, community individuals were only occasionally named as potential supports but more often named as problems. For example, there were several instances of neighbors being discussed as nosy, bad influences, or wanting to do harm.

Cultural dynamics were rarely—but effectively—referenced by counselors. For example, one counselor skillfully encouraged uptake of communication skills by emphasizing that respect is not to be viewed as a Western idea being imposed but something that has always been important to the Nandi people (see Table 4).

 Most instances of community resources were TP-promoting, with counselors using them to encourage families’ active solution-making. Sometimes, these exhortations crossed over into telling families what to do—such as going to church—or they became judgmental. Only occasionally did counselors’ practices become harmful, such as one counselor giving too many identifying details about a child’s friend (not a participant) seeking HIV testing and counseling. In another instance, the counselor’s discussion of a potential bursary seemed to over-promise this solution for a family. Finally, in the most extreme example, one counselor strongly exhorted a man to return to church, which turned into an argument, with the father stating that the counselor might as well “tie [him] up” to take him to church (Family 11). These instances were addressed in-depth during supervision, raising awareness for the counselor about negative implications and training on strategies for repairing or clarifying with the family.

### Examples and role models

Counselors frequently offered examples of people who were in or had gone through similar situations as families. Examples were mostly positive or success stories, but there were also negative examples demonstrating risks in order to warn families. Examples usually included actual, known people whom the counselor and/or family interacted with in real life, but there were also references to well-known athletes or politicians, as well as hypothetical or generic descriptions of people. Some counselors gave examples from previous families they had counseled (without mentioning their identities), perhaps to establish their counseling expertise or instill hope.

Counselors usually gave positive examples to motivate families and show them that improvement was possible, while encouraging them to put in the necessary effort. As seen in the quotation in Table 4, counselors also used examples to validate families’ experiences as shared: almost all counselors at least once explained that they knew someone in the same situation as the family member or entire family. The above example is particularly effective because it includes the positive outcome alongside an emphasis on the process and work involved. One counselor effectively elicited positive examples from families, asking for specifics about what strategies those families use.

In contrast, positive examples were less effective when they overstated potential outcomes for the family or focused only on the outcome without describing the effort or strategies for achieving change:

*Look at someone like Asbel Kiprop [an Olympic runner from Kenya]. Do you know that he used to carry water for someone who had a hotel while doing his training? At one point, his mother was even locked out because she didn’t have money to pay rent. His father was a drunkard […] Kiprop ran, he won his first race, and that family changed. So don’t say that your family cannot change* (Family 1).

Additionally, counselors’ positive example/role model stories were sometimes long and confusing, disrupting TP strategies. One counselor provided an example of how he successfully helped someone, but the story consisted of disjointed anecdotes. At other times, counselors described themselves as advising others’ children directly rather than helping the parents to do so, or suggested specific solutions rather than eliciting them. For instance, one counselor provided an example of a woman who used prayer as the sole strategy for coping with her husband’s alcohol use and concluded, “*In the same way, you can also pray for your husband and he will change too”* (Family 1). While solutions were usually appropriate, they should instead have been developed by families themselves.

Counselors also gave negative examples to show parents and children what problems they might encounter or what could happen if they do not improve. Sometimes these were gentle warnings or were presented as contrasting with the client to validate and motivate them: “*I am telling you [child] that you will succeed first because of your behavior, the good behavior. There are children who cannot listen to advice I am saying*” (Family 1). The husband–wife counseling team effectively used generic scenarios of people facing similar problems to clarify the family’s situation:

*There is this student at school who always thinks that he/she will fail the exams: ‘I cannot do my exam well; I will fail.’ There is the other [student] saying, ‘Even though it is difficult, I can read and pass.’ […]Tell me using the example, which one do you have?* (Family 8)

However, most negative examples had a threatening, foreboding tone, such as warning children about being drugged, becoming pregnant, or acquiring HIV.

Most other negative examples revolved around alcoholism and related judgment from community members. These included stories of people passed out on the road from drinking, where they could have been robbed or injured, and of people who were hospitalized. Negative examples of alcohol use were usually targeted towards male caregivers and occasionally towards adolescents. One counselor described a woman who regretted reporting her husband after he was taken to jail to discourage another woman from reporting her husband. These heavy-handed strategies contrast with more effective descriptions of counselors’ own journeys with alcoholism (see “Self-disclosure”). While almost all counselors employed Examples, two counselors accounted for most intervention-disrupting uses of this ad-hoc adaptation.

### Self-disclosure

Self-disclosure refers to counselors sharing about their own lives and experiences. This was one of the least commonly used practices but was used across the widest range of families. Self-disclosure was also the most intervention-aligned practice, aligning with intervention goals nine times more often than interrupting them. Counselors described real scenarios or behaviors, including problem drinking, relationships within their own families, and their behaviors and emotions as a child. Counselors sometimes used hypothetical examples involving themselves to show how they would handle a situation comparable to what the family had been experiencing.

For the most part, self-disclosure was used in a way that promoted therapeutic goals. For example, counselors used self-disclosure to validate or normalize participants’ feelings and challenges. One counselor said to a father, “*It is normal to feel bad when the child does something wrong to us. I think that time when you saw my adolescent [running away] I was also feeling bad”* (Family 10). Several other self-disclosure examples likewise related to behavior of counselors’ children or their methods of discipline. One counselor extended normalizing self-disclosure to use of the counseling program (see Table 4). Counselors also used self-disclosure to relate to children, including one counselor who disclosed extreme distress and homicidal thoughts as a child.

Self-disclosures aimed at validation were often used to model active solutions and TP-consistent behaviors. For example, one female counselor responded to a mother’s marriage frustrations by connecting them not to only her own but also to their family history:

*Let us remember our grandmothers and mothers persevered; that is why they are still living. In marriage life, challenges must be present […]Marriage life is persevering so that we can take the path that our parents took. If you talk with your grandmother or your mother, she will tell you that she also persevered to be the way she is now.* (Family 4)

Further, self-disclosures were sometimes used to promote communication. For example, one counselor shared their own story of misbehaving as a child and then encouraged the mother and father to do the same to help them reflect and relate to their child. Counselors also used self-disclosure to help spouses empathize with each other, sometimes by reframing how they understood their spouse’s behaviors, for example:

*Wife: But he should at least try to talk; he should not keep quiet so much that even when our child sits next to him, he/she feels like going away because of him keeping quiet.*

*Counselor: That is true. I do also keep quiet sometimes, not that I am thinking about anything bad, but maybe I have my thoughts somewhere else, but whenever someone talks to me, I will also talk. So it is important that you understand him because we are created differently.* (Family 4)

The husband–wife counseling team often presented themselves as hypothetical examples, talking through how they would communicate or face a challenge. Sometimes this strategy was effective, such as when they role-played how they would handle their children being sent home for lack of school fees and there not being food in the home. Other times, their examples seemed hopeless, were presented in long lectures, or attempted to build empathy and understanding but instead began to shift into implied judgment or subtle criticism. For example, the pair scolded a husband for not returning home for meals:

*Counselor 1: For my wife, if I will be going out without eating her food, I will cause her problems. First, she will feel that I hate her; secondly, she will think there is a place I eat, and thirdly the children will ask her why their father doesn’t eat food at home.* (Family 4)

Similarly, other counselors’ efforts at self-disclosure were sometimes less consistent with the intervention goal of generating active solutions, though without becoming harmful. For example, in several instances, counselors self-disclosed alcoholism or financial stressors, but they attributed changes to God:

“*I was a drunkard. I could take alcohol throughout the day and by around 2-3pm, the drunkardness in me could be over, and I would usually start thinking of whom I may have done something wrong to. I would ask God to just help me stop taking alcohol […] God helped me. Don’t you think he can help you too?*” (Family 1)

In two instances, self-disclosure was used in ways that directly contradict therapeutic goals. In one family, the wife complained that her husband comes home drunk and reeking of alcohol, so she chooses to sleep with the children. In response, the male counselor lectured her on the importance of having sex, framed around his own expectations about sex in his marriage (see “Religion”, above). During a module including promotion of non-violent discipline, another counselor disclosed having been beaten as a child and later said “*I thanked God that she did what she did”* (Family 18).

### Variation among counselors

As indicated above, counselors varied in their use of ad-hoc adaptations. They were employed far more often by male counselors (including the male in the couple who counseled together). While roughly twice as many transcripts come from male or paired counselors, they accounted for almost 10 times as many ad-hoc adaptations. One female counselor used only a single ad-hoc adaptation across all transcripts: a problematic use of self-disclosure. The other two female counselors were more likely to employ ad-hoc adaptations in intervention-aligned ways compared to their male counterparts (79% vs 48%, respectively).

## Discussion

In this study, we characterized ad-hoc adaptations used by Kenyan lay counselors delivering a family therapy intervention and evaluated adaptations’ alignment with therapeutic goals. Adaptations tended to promote interventions goals (49%) or were neutral (39%), though in 12% of instances, they interrupted therapeutic goals. Counselors in this study generally had high fidelity [44], and ad-hoc adaptations largely seemed focused on improving acceptability and enhancing therapeutic goals (e.g., validation, family-generated solutions), rather than incorporating new goals.

The degree of flexibility granted to lay counselors in delivering Tuko Pamoja is greater than most interventions, to the point that ad-hoc adaptations are not even possible within 4 of the 7 categories in Aarons et al.’s [1] taxonomy (process: order, dosage/intensity, addressing urgent concerns; content: excluding parts of EBP). As a result, the ad-hoc adaptations we identified concern presentation of materials and supplementing information to model materials.

The content of ad-hoc adaptations often focused on explanatory models, metaphors, and examples to communicate therapeutic content in a culturally appropriate way, which aligns with the types of changes made in planned adaptations [8, 24]. The high prevalence of religious content in this study is not surprising, since most counselors were religious leaders. Such content has strong potential for deeply culturally relevant material. Importantly, counselors did not appear to insist on discussing faith when families did not want to do so. Instead, families often raised these themes themselves. It is possible, though, that families felt this is what counselors wanted to hear.

While we could not evaluate effectiveness of the ad-hoc adaptations identified here, examining their alignment with intervention goals is one step towards answering the key question of how adaptions might affect fidelity [1]. Compared to other studies of ad-hoc adaptations, we found relatively few instances of disrupting intervention goals (e.g., [38]). Interestingly, we found an inverse relationship between how often specific ad-hoc adaptations were used and how aligned they were with therapeutic goals. Perhaps the most familiar content for adaptations (e.g., Religion, Examples) is also most susceptible to uses that interrupt therapeutic goals. Rather than discouraging use of such content, we advocate training and supervision that promote appropriate incorporation of cultural and contextual content. Our findings could also inform additions to the manualized intervention by promoting use of specific effective strategies (e.g., specific Metaphors or Self-disclosure).

Even where additions to manualized content are themselves good or neutral, these adaptations must be closely evaluated and placed within the context of taxonomies of adaptations [1]. For example, the act of addition sometimes undermined the therapeutic process, such as when counselors shared relevant morals through long-winded stories that took away time from families developing solutions. We join those who have called for incorporating ad-hoc adaptation into lay counselor training [5, 38]. Through problem-based examples, counselors could practice integrating ad-hoc adaptations in a way that maximizes benefits and minimizes potential interruptions to core content. Additionally, supervisors should elicit ways that counselors incorporate ad-hoc adaptations and provide ongoing feedback to ensure intervention alignment.

Our strategy for identifying and evaluating ad-hoc adaptations could be translated to other settings. Our approach was likely more time-consuming yet provided more detail regarding ad-hoc adaptations compared to other studies [5, 54]. Researchers and practitioners interested in studying ad-hoc adaptations must balance these trade-offs in selecting an approach, which will be influenced by time, personnel, and other resources, as well as the level of detail that is most useful given how results might be used. It may be that this more in-depth process will be useful in initial pilot studies of an intervention when it is early enough in the process to use results to revise intervention content. It is possible that ad-hoc adaptations by lay counselors could inform improvements to increase the cultural and contextual fit of a program.

In the future, the ad-hoc adaptations described in this study should be evaluated in relation to implementation and effectiveness outcomes to systematically identify which practices were helpful and harmful. Ideally, ad-hoc adaptations should be studied prospectively. Systematically considering ad-hoc adaptations during trainings could also be used as an implementation strategy to rapidly incorporate cultural and contextual content when scaling up interventions like Tuko Pamoja to other settings.

There are notable limitations to this study. Since the therapy sessions were translated from Kiswahili to English, there is a chance that some meaning was lost or misunderstood. Formal data analysis was led by US researchers and informed by discussions with Kenyan supervisors. There is a chance that some practices were more or less likely to stand out as cultural or community-based practices to both US and Kenyan team members. To some extent, the quantification of intervention alignment reflects how we segmented code segments in ad-hoc adaptation coding. However, our process of applying a code once to each continuous text segment where that theme arose followed standard procedures. The supervision process likely altered counselors’ ad-hoc adaptations throughout implementation, perhaps reducing the number of TP-interrupting adaptations; without intervention via supervision, there may have been more such problematic adaptations.

## Conclusion

The study’s evaluation of ad-hoc adaptations used by lay counselors in delivering family therapy in Kenya shows the diversity of novel content, which was largely used in alignment with intervention goals. Early and consistent attention to ad-hoc adaptations can support the balancing of contextual fit and fidelity to interventions in cross-cultural work.

## Data Availability

Data are available upon reasonable request to the first author.

## Abbreviations

EBP: Evidence-based practice
TP: Tuko Pamoja (Swahili: “We are Together”)

## Footnotes

1 Many of these examples are similes, personification, or other literary devices, but we use the term metaphor throughout for simplicity’s sake.
